# HCFC-123-induced toxic hepatitis and death at a Korean fire extinguisher manufacturing facility: a case series

**DOI:** 10.1186/s40557-018-0231-2

**Published:** 2018-03-28

**Authors:** Mu Young Shin, Jong Soo Park, Hae Dong Park, Jihye Lee

**Affiliations:** 1 Occupational Safety and Health Research Institute, Korea Occupational Safety and Health Agency, Ulsan, Republic of Korea; 2 Occupational Safety and Health Research Institute, Korea Occupational Safety and Health Agency, 478, Munemi-ro, Bupyeong-gu, Incheon, 21417 Republic of Korea

**Keywords:** 2,2-dichloro-1,1,1-trifluoroethane, HCFC-123, Toxic hepatitis, Drug-induced liver injury, Fire extinguisher manufacturing facility

## Abstract

**Background:**

Exposure to sustained high concentrations of HCFC-123 is known to be hepatotoxic. We report two simultaneous cases of toxic hepatitis related to exposure to 2,2-dichloro-1,1,1-trifluoroethane (HCFC-123), a common refrigerant, at a Korean fire extinguisher manufacturing facility.

**Case presentation:**

Patients A and B were men aged 21 and 22 years, respectively, with no notable medical histories. They had recently started working for a manufacturer of fire extinguishers. During the third week of their employment, they visited the emergency center of a general hospital due to fever, lack of appetite, and general weakness. At the time of their visit, they were suspected as having hepatitis due to elevated aspartate aminotransferase (AST), alanine aminotransferase (ALT), alkaline phosphatase (ALP), gamma-glutamyl transferase (GGT), and total bilirubin levels and were hospitalized. However, as their condition did not improve, they were moved to a tertiary general hospital. After conservative treatment, one patient improved but the other died from acute hepatic failure. Assessments of the work environment showed that the short-term exposure levels of HCFC-123 for valve assembly processes were as high as 193.4 ppm. A transjugular liver biopsy was performed in patient A; the results indicated drug/toxin-induced liver injury (DILI). Given the lack of a medical history and the occupational exposure to high levels of HCFC-123, a hepatotoxic agent, the toxic hepatitis of the workers was likely related to HCFC-123 exposure.

**Conclusions:**

Work environment assessments have not included this agent. To the best of our knowledge, we are the first to report a case of death related to HCFC-123-induced liver damage. Our findings suggest that exposure standards and limits for HCFC-123 must be developed in Korea; work environments will have to be improved based on such standards.

## Background

2,2-dichloro-1,1,1-trifluoroethane (HCFC-123; CAS registry number 306–83-2), often known as Freon™123, is a volatile liquid with a boiling point of 27.6 °C; it is easily gasified at room temperature [1]. As HCFC-123 has a low ozone depletion potential (ODP) when compared to CFC-11 (ODP; 0.02, which is about 1/50 of that of CFC-11), it is often used as an alternative to CFC-11 [2]. HCFC is short for hydrochlorofluorocarbons and indicates compounds that include hydrogen, carbon, fluorine, and chlorine. HCFC-123 is mainly used as a commercial and industrial refrigerant and often as a heat-transfer fluid, foam-blowing agent, or solvent [1, 3]. It is classified as a fire extinguishing agent through cooling and dilution. As HCFC-123 has a fair extinguishing ability and low toxicity and does not leave residues after fire extinction, it is often used as a recharging material for fire extinguishers [4].

Inhalation of high concentrations of HCFC-123 in humans can lead to nervous system depression, symptoms such as dizziness, headache, confusion, incoordination, and loss of consciousness, and can even result in cardiac arrest. It may cause irritation to the eyes [3]. Exposure to sustained high concentrations of HCFC-123 is known to be hepatotoxic. Hepatotoxicity has been shown in guinea pigs in 1994 [5]. HCFC-123 produces trifluroacetyl halide, which causes hepatoxicity; P58 and P450 2E1 autoantibodies were found in workers who were exposed to HCFC-123/− 124. It suggests that there is a possibility that immune responses may also act on the mechanism of hepatotoxicity. 1-bromo-1-chloro-2,2,2-trifluoroethane (halothane) is known to cause serious liver damage in susceptible individuals after inhalation. HCFC-123 has a similar structure as halothane and is metabolized by the same pathway. Moreover, the P58 and P450 2E1 autoantibodies are found in patients with halothane hepatitis. This suggests that HCFC-123 may cause liver damage similar to halothane hepatitis [6, 7].

Several cases of liver damage related to HCFC-123 exposure have been reported (one case in Belgium in 1996, two cases in Japan in 1997 and 1999, one case in the US in 1997, and one case in Korea and one case in Hong Kong in 2010) [6–11]. The Belgian case was related to the leakage of HCFC-123 during the boring of plastic tubes. The patient’s medical records indicated the following results: aspartate aminotransferase (AST) 607 U/L, alanine aminotransferase (ALT) 1298 U/L, alkaline phosphatase (ALP) 303 U/L, and gamma-glutamyl transferase (GGT) 106 U/L. There were no further hepatitis cases after the air-conditioning system was repaired [6]. In 1997, 9 Japanese workers were exposed to HCFC-123 at a refrigerant sealing facility due to a leakage; the workplace had no local ventilation system. Whereas 5 patients had normal bilirubin levels and only a minimal rise in AST and ALT levels, the AST and ALT levels of the remaining 4 workers rose to 226–822 IU/L and 297–1715 IU/L, respectively. After conservative treatment, their liver tests returned to normal, and the symptoms improved [8]. In 1999, a dry cleaner of 8 years at a bridal section in a hotel in Japan contracted hepatitis. She showed the following liver enzyme levels: AST 2125 IU/L, ALT 2391 IU/L, GGT 294 IU/L, and total bilirubin 8.5 mg/dL. On the first day of her return to work after the symptoms improved, she started experiencing a relapse of the symptoms. At this time, the blood tests showed the following results: AST 1354 IU/L, ALT 1604 IU/L, GGT 193 IU/L, and total bilirubin 2.6 mg/dL. The patient’s condition improved with conservative treatment, and she was discharged. At work site, the geometric mean concentrations of HCFC-123 were 1355 and 1986 ppm [9]. In the US, the case involved workers using HCFC-123 as a degreaser. Personal samples that were taken on the day after introducing HCFC-123 to the electronic component processing factory showed exposure levels of 24–480 ppm; area samples showed levels of 18–180 ppm. Samples taken after liver damage was identified showed exposure levels of 5–12 ppm. Two patients showed maximum AST and ALT levels of 1370 mu/mL and 2675 mu/mL, respectively, or 956 mu/mL and 3001 mu/mL, respectively. All patients eventually recovered [10]. In Korea, a worker was exposed to refrigerant gas while exchanging refrigerant fluids due to a leakage during refrigerator maintenance. He visited the hospital due to fever and general weakness, and blood examinations revealed the following: AST 536 IU/L and ALT 441 IU/L; he later recovered [11]. Last, a case in Hong Kong in 2010 involved the leakage of HCFC-123 used as refrigerant. The patient showed the following liver test results: AST 2173 IU/L, ALT 1898 IU/L, and serum bilirubin 62 μmol/L; elevated liver test was also showed in 4 other individuals who agreed to be examined. All individuals recovered after they were no longer exposed to HCFC-123. In this case series, is noteworthy that, unlike in past cases, a death occurred, and this death occurred in a fire extinguisher manufacturing facility. This suggests that HCFC-123 can be fatal in humans.

While there are no antidotes for the liver damage related to HCFC-123 exposure, conservative treatment generally leads to recovery [7].

## Case presentation

### Patient a (21-year-old man)

#### Chief complaints

Fever, chills, and myalgia.

#### Past medical history

Hypertension/Diabetes mellitus/Tuberculosis/Hepatitis (−/−/−/−).

Social history: non-smoker, non-drinker.

Medications: Finasteride, from January 2017.

#### Present illness

Patient A showed symptoms of fever, chills, and myalgia starting on August 12. The symptoms continued, and the patient visited the emergency room of a general hospital one day later. While he underwent conservative treatment, he showed over 2000 IU/L of AST and ALT and no signs of improvement; therefore, he was moved to a tertiary general hospital, together with patient B. Laboratory examinations at the tertiary general hospital on August 15 indicated clearly elevated liver enzyme levels (AST 2491 IU/L, ALT 2355 IU/L, GGT 102 IU/L, and total bilirubin 3.4 mg/dL). Conservative treatment was provided. Anti-HAV IgM, HBsAg, HBcAb IgM, anti-HBs, and anti-HCV were negative, while anti-HAV IgG was positive, likely due to a past type A hepatitis vaccination. He tested negative for human immunodeficiency virus. Because two individuals working at the same facility showed symptoms concurrently, an occupational disease was suspected. To obtain an accurate occupational history, patient interviews and material safety data sheet checks were conducted. It was suspected that HCFC-123 was the most likely cause of hepatotoxicity. The patient underwent a transjugular liver biopsy that indicated portal and panlobular infiltration of mixed inflammatory cells (abundant eosinophils) with lobular disarray, multifocal spotty and confluent necrosis, mild bile duct damage, and minimal macrovesicular and microvesicular steatosis; these findings were considered suspicious for DILI as they demonstrated patterns of acute hepatitis. The patient’s liver enzyme levels rose to AST 3108 IU/L and ALT 3265 IU/L but then decreased on August 21 to AST 117 IU/L and ALT 662 IU/L. Subsequently, he was improved and moved to a general hospital. The progression of patients A and B were shown in Tables 1 and 2.

**Table 1:** Progression of patient A^a^

	Day 3	Day 4	Day 6^b^	Day 9	Reference range
AST (IU/L)	2491	3108	302	117	0–40
ALT (IU/L)	2355	3265	1708	662	0–40
ALP (IU/L)	125	139	144	165	30–115
Total bilirubin (mg/dL)	3.4	3.3	4.0	1.9	0.2–1.2
GGT (IU/L)	102	96	–	–	11–63

*ALT* alanine aminotransferase, *ALP* alkaline phosphatase, *AST* aspartate aminotransferase, *GGT* gamma-glutamyl transferase

^a^Discharged and returned home on day 10

^b^GGT was not measured on days 6 and 9

**Table 2:** Progression of patient B^a^

	On admission	Day 3^b^	Day 4	Day 6	Day 11	Reference range
AST (IU/L)	1551	13,537	16,152	5557	352	0–40
ALT (IU/L)	1259	7115	8245	4048	409	0–40
ALP (IU/L)	74	85	170	361	341	30–115
Total bilirubin (mg/dL)	1.59	3.6	6.8	10.6	34.1	0.2–1.2
GGT (IU/L)	154	155	146	100	39	11–63

*ALT* alanine aminotransferase, *ALP* alkaline phosphatase, *AST* aspartate aminotransferase, *GGT* gamma-glutamyl transferase

^a^Pronounced dead in the early morning of day 12

^b^The patient was transferred to a different hospital on day 3

### Patient B (22-year-old man)

#### Chief complaints

Fever, anorexia, and general weakness.

#### Past medical history

Hypertension/Diabetes mellitus/Tuberculosis/Hepatitis (−/−/−/−).

Social history: smoker (4 pack-years), social drinker (4 standard drinks per week).

#### Present illness

Patient B reported fever, anorexia, and general weakness starting on August 11. As the symptoms continued, he visited the emergency room of a general hospital 2 days after the onset of the symptoms. Upon admission, laboratory examinations indicated thrombocytopenia and the following findings: hemoglobin 17.4 g/dL, leukocytes 7500/μL, platelets 94,000/μL; blood urea nitrogen (BUN) 17.5 mg/dL, and creatinine 1.14 mg/dL. Elevated liver enzyme levels were found (AST 1551 IU/L, ALT 1259 IU/L, GGT 154 IU/L, and total bilirubin 1.59 mg/dL). The prothrombin time showed an international normalized ratio (INR) of 1.35, indicating slight prolongation. The serology tests that were conducted due to the high liver enzyme results showed that the patient tested negative for anti-HAV IgM, anti-HAV IgG, HBsAg, HBcAb IgM, anti-HBs, and anti-HCV. Routine urinalysis showed signs of proteinuria. The patient was hospitalized and underwent conservative treatment. However, as he did not show any improvements but presented with elevated liver enzyme levels, thrombocytopenia, and acute renal failure, he was moved to a tertiary general hospital 2 days later. The laboratory examinations at the tertiary general hospital showed the following: AST 13,537 IU/L, ALT 7115 IU/L, GGT 155 IU/L, and total bilirubin 3.6 mg/dL. The patient underwent tests to identify the cause of the hepatitis and to rule out infections. IgM anti-HEV and IgG anti-HEV were both negative; thus, infection by the hepatitis E virus was considered unlikely. He also tested negative for human immunodeficiency virus, cytomegalovirus, Epstein-Barr virus, herpes simplex virus, and varicella-zoster virus; thus, a recent infection was excluded. IgG for measles and mumps was positive, and IgG for rubella was in the borderline positive, which was likely caused by a recent measles, mumps, and rubella (MMR) vaccination. Antibody tests for the Hantaan virus, Leptospira, Rickettsia typhi, and Rickettsia tsutsugamushi were negative. A stool culture did not isolate salmonella, shigella, or vibrio species, and no growth was detected in a blood culture. Tests for autoimmune hepatitis showed positive antinuclear antibody staining with a speckled pattern at 1:40. Anti-mitochondrial, anti-neutrophil cytoplasmic, smooth muscle, and anti-LKM antibodies were all negative. The patient’s serum IgG concentration was 530 mg/dL, which is below the reference range (700–1700 mg/dL). Therefore, the patient likely had no autoimmune hepatitis. No liver biopsy could be performed as the patient had a poor general condition due to a cerebral hemorrhage. However, a transjugular liver biopsy could be performed in his co-worker (patient A) who was co-hospitalized; these results indicated drug/toxin-induced liver injury (DILI). Therefore, it appears likely that patient B also had DILI. On August 17, he showed a lowered level of consciousness, hypersensitivity, and prothrombin time prolongation; therefore, he was moved to the intensive care unit (ICU) under suspicion of acute fulminant hepatitis and underwent continuous renal replacement therapy and Molecular Adsorbent Recirculating System (MARS®) therapy. There is no specific treatment for HCFC-123-induced toxic hepatitis. Although an occupational disease was suspected, and the patient was referred to an occupational and environmental medicine physician, the disease progression could not be slowed. The patient did not respond to conservative treatment. Seven days later, on August 24, the patient died from acute hepatic failure while undergoing treatment at the ICU.

#### Occupational history

Patient A began work at the fire extinguisher manufacturing facility on July 26; his symptoms of fever, chills, and myalgia began 17 days later on August 12. Patient B started working at the same facility on July 27; his symptoms of fever, anorexia, and general weakness began 15 days later on August 11. The company manufactures fire extinguishers and gas sprayers and uses HCFC-123, HFC-125, HFC-236FA, and loaded stream. HCFC-123 charging involves a simple procedure, and both, contract workers and new employees are in charge of this process. The fire extinguisher manufacturing process consists of component assembly prior to charging, charging of the HCFC-123 fire extinguishing agent, packing, and consignment. Typically, the patients A and B were tasked with component assembly, although they were often involved in the entire manufacturing process. The HCFC-123 charging process involves filling the storage tank with HCFC-123, placing the empty fire extinguisher on the conveyor belt, charging the empty extinguisher cans with HCFC-123, assembling the valves and seal rings on the fire extinguishers, nitrogen charging, and manual sealing tests. Filling the empty fire extinguisher cans with HCFC-123 is automated as they move across the conveyor belt; the charging time per extinguisher is approximately 20 s. During the first two weeks of employment, the patients A and B were not tasked with HCFC-123 charging duties. Patients A and B were tasked with HCFC-123 charging for about 5 h split up into 3 days between August 9 and August 11. Patient A was tasked with nitrogen charging, whereas patient B was involved in the valve assembly process.

#### Exposure assessment

The work environment was assessed for the time-weighted average (TWA), short-term, and area exposure levels of HCFC-123 (Tables 3 and 4). The TWA exposure levels were 19.1–20.9 ppm; they did not surpass the exposure limits set by the American Industrial Hygiene Association (AIHA; 50 ppm) but exceeded those set by the Japan Society for Occupational Health (JSOH) and Honeywell (10 ppm). No exposure limits for HCFC-123 have been set in Korea to date. The short-term exposure levels across 15 min of measurement were 149.3–193.4 ppm for the valve assembly process and 114.6–114.9 ppm for the nitrogen charging process. As there are no exposure limits for short-term exposure, we used 3–5 times the TWA exposure limits, according to the American Conference of Governmental Industrial Hygienists (ACGIH) peak exposures (150–250 ppm for AIHA and 30–50 ppm for JSOH and Honeywell). The short-term exposure levels during the valve assembly portion appeared to be significant. The area exposure levels revealed HCFC-123 concentrations of 58.3–187.2 ppm for the valve assembly process and 75.5–112.3 ppm for the nitrogen charging process.

**Table 3:** Eight-hour time-weighted average exposure level assessment for HCFC-123

Worker	Raw material pumping		Automatic charge line			Exposure standard
	Exposure concentration	Time measured	Exposure concentration	Time measured	TWA	
	(ppm)	(min)	(ppm)	(min)	(ppm)	
1	20.7	73	91.6	89	20.1	50 ppm (AIHA) 10 ppm (JSOH)
2^a^	–		115.6	87	20.9	
3	–		71.8	131	19.6	
4	–		69.5	132	19.1	

*AIHA* American Industrial Hygiene Association, *JSOH* Japan Society for Occupational Health, *TWA* time-weighted average

^a^A hyphen means that it was not measured

**Table 4:** Short-term and area exposure level assessment of HCFC-123

	Valve assembly		Nitrogen charging		Exposure standard
	Exposure concentration	Time measured	Exposure concentration	Time measured	
	(ppm)	(min)	(ppm)	(min)	
Short-term exposure	149.3–193.4	15	114.6–114.9	15	None
Area exposure	58.3–187.2	75–90	75.5–112.3	90–92	

In both, short-term and area exposure assessments, HCFC-123 levels were highest for the valve assembly process, likely because this process was in close physical proximity (about 1 m) to the automatic HCFC-123 injector. In contrast, nitrogen charging was performed about 3 m away from the automatic HCFC-123 injector.

## Discussion and conclusions

We report two cases of liver damage due to exposure to HCFC-123 in workers working at a fire extinguisher manufacturing facility in Korea. Patient B died, whereas patient A recovered. Whereas patient B performed valve assembly during the HCFC-123 charging process, patient A was tasked with nitrogen charging. HCFC-123 exposure levels were highest for the valve assembly process, which is in closer proximity to the automatic HCFC-123 injector, in both, short-term and area exposure assessments. Therefore, patient B was likely exposed to higher levels of HCFC-123 than patient A, and this higher exposure likely led to his death. The workers were not familiar with the risks related to HCFC-123, which may have affected their behavior. An automatic charge line was implemented at the facility in April of the same year, followed by an increase in production, making indirect exposure to HCFC-123 possible. Therefore, health examinations were conducted for all co-workers in the same workplace. In these health examinations, 6 of 15 co-workers showed abnormal liver tests. Moreover, a further investigation revealed one patient who was suspected of having toxic hepatitis in June of the same year.

No exposure limits for HCFC-123 have been set in Korea. In the US, the AIHA Workplace Environmental Exposure Levels (WEEL) Committee set the TWA exposure limit at 50 ppm, based on a 40-h workweek [12]. In contrast, JSOH and Honeywell set the limit 10 ppm [13, 14]. As this is a five-fold difference, it appears likely that the background of setting these limits differs greatly. In a 1997 case of HCFC-123-induced hepatotoxicity in the US, the estimated building-wide 8-h TWA exposure was 10–35 ppm [10]. According to best estimates, the 8-h TWA in the work area was 50–163 ppm. The patient in this case experienced toxic hepatitis, with AST levels of 1370 mu/mL and ALT levels of 2675 mu/mL [10]. Shin et al. evaluated 4 Korean factories manufacturing industrial air conditioners in 1998. The geometric mean 8-h TWA levels of HCFC-123 were 2.2 ppm (0.89–4.2 ppm) and 32.5 ppm (4.9–113.9 ppm) in two factories, whereas the other two factories reported lower levels. In factory B, the short-term HCFC-123 concentration was 192.4 ppm (11.3–1065 ppm) in the refrigerant charging and degassing facilities. Liver tests that were conducted for all exposed workers were normal. This study reported that, when the 8-h TWA was < 32.5 ppm for HCFC-123, no hepatotoxicity was found [15].

To date, it has been thought that hepatotoxicity can be avoided if the 8-h TWA exposure limit of 50 ppm set by the AIHA WEEL Committee is met and safety equipment is worn by workers. However, in this case the 8-h TWA for the workers was 19.1–20.9 ppm. As Korea has set no exposure standards for HCFC-123, defining such exposure standards and limits should be prioritized.

The diagnostic criteria for DILI are an ALT level of > 5 times the upper limit of normal (ULN), or an ALP level of > 2 times the ULN, or an ALT level of > 3 times the ULN with a bilirubin concentration of > 2 times the ULN [16]. There are three conditions for diagnosing occupational toxic hepatitis: 1) the occurrence of liver damage after occupational exposure, 2) liver enzymes elevated more than twice the ULN, and 3) other causes were ruled out [17]. In the cases reported herein, all three conditions were satisfied. In cases of toxic hepatitis, the toxic agents can be absorbed through inhalation, oral consumption, and through the skin. Hepatotoxins can be divided into intrinsic toxins and host idiosyncrasy, depending on their mechanism. Intrinsic toxins lead to dose-dependent liver damage, with quick surfacing of symptoms. Host idiosyncrasy is dose-independent; it depends on individual sensitivity, and liver damage may or may not occur [18]. HCFC-123 is considered to be an intrinsic toxin [19]. However, there may also be host idiosyncrasy. Because host idiosyncrasy may be involved in hepatotoxicity, current exposure standards may not be able to prevent hepatotoxicity. Therefore, it is necessary to continuously monitor workers’ health through special health examinations and to establish exposure standards.

HCFC-123 is used globally, especially in the form of HCFC-BLEND B by mixing CF_4_ and argon. HCFC-BLEND B consists of > 93% HCFC-123 [20]. When using a fire extinguisher, a person can be exposed to HCFC-123. Meridian Research, Inc. measured the airborne levels of HCFC-123 in firemen with breathing aids who used fire extinguishers containing 93% HCFC-123. The breathing-zone levels of HCFC-123 across the entire experiment were 7–870 ppm. When hand-held fire extinguishers were utilized within aircraft hangars, the breathing-zone concentration while spraying was 20 ppm. When larger semi-portable fire extinguishers were used, a breathing-zone concentration of 180–300 ppm was observed while spraying [21, 22]. The Midwest Research Institute reported breathing-zone levels of HCFC-123 of 0.2–5.4 ppm after a few minutes of using 45 kg or 59 kg fire extinguishers. HCFC-123 levels in the plume were 0.18–180 ppm [23]. In summary, when a smaller fire extinguisher is used in a well-ventilated area, the breathing-zone concentration likely has no health effects. Under the Montreal Protocol, the use of HCFC-123 will cease in most countries between 2020 and 2030 [1].

Previous cases of HCFC-123-induced liver damage were related to HCFC-123 being used as a refrigerant or solvent; moreover, there have been no reported deaths so far. In this report, HCFC-123 was used as a fire extinguishing agent by workers at a fire extinguisher manufacturing facility. It has been reported that HCFC-123-induced liver damage can be effectively treated with conservative treatment; however, a death occurred in our case. The concentrations of HCFC-123 were not higher in this case when compared to past cases. There is a possibility that host idiosyncrasy might affect the liver in patient B.

As HCFC-123 is widely used as a fire extinguishing agent, its safety should be reevaluated. To reduce the occupational risks of HCFC-123 exposure, it is crucial to maintain a health & safety management system, foster technological innovation, adhere to regulations, and implement effective employee oversight strategies [24].

In conclusion, there have been only few cases of liver damage from HCFC-123 exposure globally. To the best of our knowledge, we are the first to report a case of death caused by HCFC-123-induced liver damage. Conservative treatment has been thought to be effective in the treatment of CFC-123-induced liver damage; however, in our case, one patient died. Unlike previous cases, exposure to HCFC-123 happened at a manufacturing facility for fire extinguishers in this case. Our findings indicate that exposure standards and limits should be set in Korea.

## Abbreviations

ACGIH: American Conference of Governmental Industrial Hygienists
AIHA: American Industrial Hygiene Association
ALP: Alkaline phosphatase
ALT: Alanine aminotransferase
AST: Aspartate aminotransferase
BUN: Blood urea nitrogen
CFC-11: Trichlorofluoromethane
DILI: Drug-induced liver injury
GGT: Gamma-glutamyl transferase
HCFC-123: 2,2-dichloro-1,1,1-trifluoroethane
ICU: Intensive care unit
INR: International normalized ratio
JSOH: Japan Society for Occupational Health
MMR: Measles, mumps, rubella
ODP: Ozone depletion potential
TWA: Time-weighted average
ULN: Upper limit of normal
WEEL: Workplace environmental exposure levels
